# Nanoparticle-mediated tumor cell expression of mIL-12 via systemic gene delivery treats syngeneic models of murine lung cancers

**DOI:** 10.1038/s41598-021-89124-4

**Published:** 2021-05-06

**Authors:** Hye-Hyun Ahn, Christine Carrington, Yizong Hu, Heng-wen Liu, Christy Ng, Hwanhee Nam, Andrew Park, Catherine Stace, Will West, Hai-Quan Mao, Martin G. Pomper, Christopher G. Ullman, Il Minn

**Affiliations:** 1 Division of Nuclear Medicine and Molecular Imaging, Russel H. Morgan Department of Radiology and Radiological Science, Johns Hopkins University, School of Medicine, Baltimore, MD 21205 USA; 2 Cancer Targeting Systems, 1188 Centre Street, Newton Centre, MA 02459 USA; 3 Department of Materials Science and Engineering, Johns Hopkins University, Baltimore, MD 21218 USA; 4 Department of Biomedical Engineering, Translational Tissue Engineering Center, Johns Hopkins University, School of Medicine, Baltimore, MD 21287 USA; 5 Institute for NanoBioTechnology, Johns Hopkins University, Baltimore, MD 21218 USA; 6 Present Address: AstraZeneca (MedImmune), One Medimmune Way, Gaithersburg, MD 20878 USA; 7 Present Address: Platform First Ltd, 1 Station Road, Sutton, Cambridge, CB6 2RL UK; 8 Present Address: Paratopix Ltd., Bishop’s Stortford, CM23 5JD UK

**Keywords:** Cancer immunotherapy, Molecular medicine

## Abstract

Treatment of cancers in the lung remains a critical challenge in the clinic for which gene therapy could offer valuable options. We describe an effective approach through systemic injection of engineered polymer/DNA nanoparticles that mediate tumor-specific expression of a therapeutic gene, under the control of the cancer-selective progression elevated gene 3 (*PEG-3*) promoter, to treat tumors in the lungs of diseased mice. A clinically tested, untargeted, polyethylenimine carrier was selected to aid rapid transition to clinical studies, and a CpG-free plasmid backbone and coding sequences were used to reduce inflammation. Intravenous administration of nanoparticles expressing murine single-chain interleukin 12, under the control of *PEG-3* promoter, significantly improved the survival of mice in both an orthotopic and a metastatic model of lung cancer with no marked symptoms of systemic toxicity. These outcomes achieved using clinically relevant nanoparticle components raises the promise of translation to human therapy.

## Introduction

Lung cancer is one of the most common and deadliest cancers with an overall 5-year survival rate for all suffers of 18% and a current cure rate of approximately 5.5%. Although the cure rate is rising slowly and overall incidences are declining, the rate of improvement has been slower than for other common cancers^1^. The discovery of checkpoint inhibitors has helped to increase long-term survival rates in combination therapy leading to an environment of rapidly changing standard of care. However, in practice, approximately 80% of newly diagnosed advanced non-small cell lung cancer patients (NSCLC) present with inoperable disease and the options for metastatic disease in lung are limited^1,2^. While the cellular origins of lung cancer are still unknown or uncertain, more than 50% of lung adenocarcinomas have identifiable mutations in critical oncogenes that potentiate tumor proliferation^3^. Targeted therapies are either limited (EGFR or ALK) or unavailable (KRAS or BRAF)^4^. In addition, lung cancers are heterogeneous with genomic and phenotypic differences not only among different patients but also within the same patient^5^. Therefore, treatments that are independent of driver mutations have broader applications when used as a monotherapy for primary lung cancer or in combination for treatment of metastases to the lung from cancers in other organs^6,7^.

While gene therapy offers huge potential in this disease area, currently no systemically delivered genetic constructs as therapeutic payloads have been approved for the treatment of cancer; and the only intratumorally delivered oncolytic virus treatment approved to date (T-Vec) is for melanoma^8,9^. Biodistribution, efficiency of transfection, and immunogenicity represent significant hurdles in the translation of these technologies into the clinic, especially for systemic parenteral delivery. Linear polyethylenimine (l-PEI) has been tested in clinical trials and is considered the ‘gold standard’ carrier for non-viral delivery of nucleic acids^10^. As is the case for virally delivered gene therapies, transfection efficiencies are low. However, unlike viral delivery, l-PEI nanoparticles are non-immunogenic, thereby permitting repeat administration^11^. Following systemic administration via the tail vein in rodents, l-PEI/DNA nanoparticles primarily locate to the lungs, liver and spleen^5^ and, therefore, it is likely that these nanoparticles can be used to deliver therapeutic genes preferentially and repeatedly to these organs. Careful formulation and consideration of the size of these non-viral nanoparticles can also potentially enable a degree of selectivity from the perspective of enhanced permeability and retention (EPR) effect of tumors^12,13^. Precise control of the size and composition of l-PEI/DNA nanoparticles is possible using novel formulation techniques, such as flash nanocomplexation (FNC)^14–16^. Another critical aspect for systemic delivery of a genetic payload is the content of unmethylated CpG sequence, which triggers acute inframammary response as an innate immune reaction^17–19^. Removing CpG sequence while maintaining functions of the genetic payload will be necessary to minimize unnecessary acute toxicity.

The expression of payload genes carried within the nanoparticle can be selectively controlled by the promoter activity. Here, we have chosen the progression elevated gene 3 promoter (*PEG-3*) promoter from the *PEG-3* oncogene, which has previously been used for tumor-specific imaging of melanoma, breast cancer, and prostate cancer cells in vivo^9–11^. The anti-tumor therapeutic used in this study is the cytokine interleukin-12 (IL-12), which has been of interest to researchers in oncology for many years. IL-12 orchestrates the activities of the innate and adaptive immune systems for destroying cancer cells and is known to exert potent anti-tumour effects primarily by stimulating the production of its downstream effector, IFN-γ, by NK cells and T lymphocytes^20^. However, its development as an anti-cancer drug has been marred by a narrow therapeutic window and severe toxicity when administered systemically in the form of a recombinant protein^20^. Therefore, the potential to express therapeutic levels of IL-12 within tumours, especially metastases, via repeated systemic administration of nanoparticles could safely harness its therapeutic effects for the treatment of cancer. In this study we tested the delivery efficiency, treatment efficacy and safety of systemically administered l-PEI/DNA nanoparticles that express IL-12, alone or in combination with other genes, Herpes Simplex Virus 1 thymidine kinase (HSV1-tk) and IL-2, in two mouse models that represent primary lung tumours and metastatic tumours.

## Results

### Functional gene payloads are expressed from the *PEG-3* promoter in vitro

We first verified the cancer-selective activity of the *PEG-3* promoter to drive expression of luciferase in human and murine cancer cell lines (Fig. S1) supporting the published data^21,22^. Following confirmation of *PEG-3* activity, interleukin 12 (IL-12) payloads, with or without additional coding sequences for Herpes Simplex Virus-1 Thymidine Kinase (HSV1-TK) or IL-2, were cloned downstream of the *PEG-3* promoter in a CpG-free plasmid (Fig. S2). A single chain IL-12 sequence was used as this had previously been shown to be active and would allow the correct stoichiometry of the p40 and p35 subunits without expression of inhibitory p40 dimer^23–25^. The activity of the *PEG-3* promoter and the expression of the payloads were verified by ELISA in a murine Lewis lung carcinoma cancer cell line (LL/2-Red-Fluc) prior to in vivo testing (Table S1).

We confirmed the functions of the expressed payloads in cancer setting. For HSV1-TK, a cytotoxicity assay was used to measure the ability of converting a prodrug ganciclovir and exhibited dose-dependent cytotoxicity (Fig. S3A). In order to demonstrate functional expression of IL-2 and IL-12, we performed a CTLL-2 expression assay. Both human and murine IL-2 can act on CTLL-2 cells and mIL-12 has also been shown to have a proliferative effect in the presence of IL-2. The results showed that undiluted culture supernatant from LL/2 cells transfected with nanoparticles expressing murine IL-12 and human or mouse IL-2, caused equivalent proliferation of CTLL-2 cells, demonstrated expression of active IL-2 and mIL-12, whereas a negative control (supernatant from cells transfected with PEG-lucia nanoparticles) had no effect (Fig. S3B). An additional functional assay was used to determine activity of IL-12 in human peripheral blood mononuclear cells (PBMCs). Cell culture supernatants from LL/2 cells transfected with nanoparticles expressing mIL-12 either alone or in combination with other human or murine IL-2 or HSV1-TK proteins showed a proliferative response from PBMCs isolated from two human donors (Fig. S3C).

### Nanoparticles with reduced CpG content diminished induction of acute inflammatory cytokines in vivo

**Figure 1:**
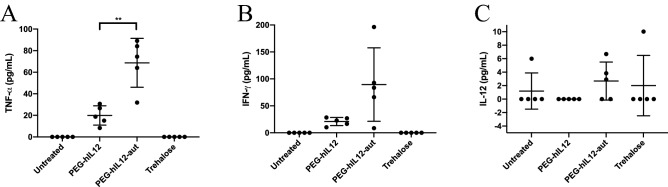
Grouped plots for cytokine stimulation in CD-1 mice. Following treatment with l-PEI/DNA nanoparticles formulated with plasmids PEG-hIL12 (43 CpGs) or PEG-hIL12-aut (152 CpGs), or 9.5% trehalose, or left untreated, the levels of endogenous TNF-α (**A**), IFN-γ (**B**) and IL-12 (**C**) were assayed. A significant difference (paired t-test, *p* < 0.01) was noted between PEG-hIL12 and PEG-hIL12-aut in the stimulation of TNF-α (**A**). There was no significant difference (paired t-test, *p* < 0.05) between PEG-hIL12 and PEG-hIL12-aut in the stimulation of IFN-γ (**B**) or endogenous murine IL-12 (**C**).

We evaluated effect on systemic administration of unmethylated CpG sequences. The activation of cytokines in vivo was assessed by monitoring levels of endogenous murine tumor necrosis factor α (TNF-α), interferon γ (IFN-γ) and IL-12 following injection of nanoparticles expressing human IL-12 (PEG-hIL12) into healthy CD-1 mice. PEG-hIL12 is a CpG-free plasmid backbone containing the *PEG-3* promoter and a sequence for single chain human IL-12 (which does not activate the rodent IL-12 receptor) and has 43 CpG sites. A plasmid incorporating the same plasmid components as PEG-hIL12 but with a CpG-containing neomycin gene and a CpG-containing human IL-12 sequence (plasmid PEG-hIL12-aut) was used as a control; this plasmid having 152 CpG sites. Analysis of blood taken 2 h post-injection showed an immediate inflammatory response to the nanoparticles following administration (Fig. 1), however, the level of TNF-α in the mice injected with PEG-hIL12 is significantly lower (*p* = 0.0021) than that in mice injected with PEG-hIL12-aut. The difference in the level of IFN-γ was also approaching significance, *p* = 0.056. Induction of murine IL-12 was negligible for both plasmids tested while a research grade plasmid with 357 CpG sequences showed a significantly higher amount of mIL-12 (Fig. S4). Therefore, reduction of the CpG content of the plasmid fulfilled the intention of reducing immediate inflammation and off-target immune effects.

### Nanoparticle-delivered luciferase plasmid with the *PEG-3* promoter was active in tumour bearing lungs

**Figure 2:**
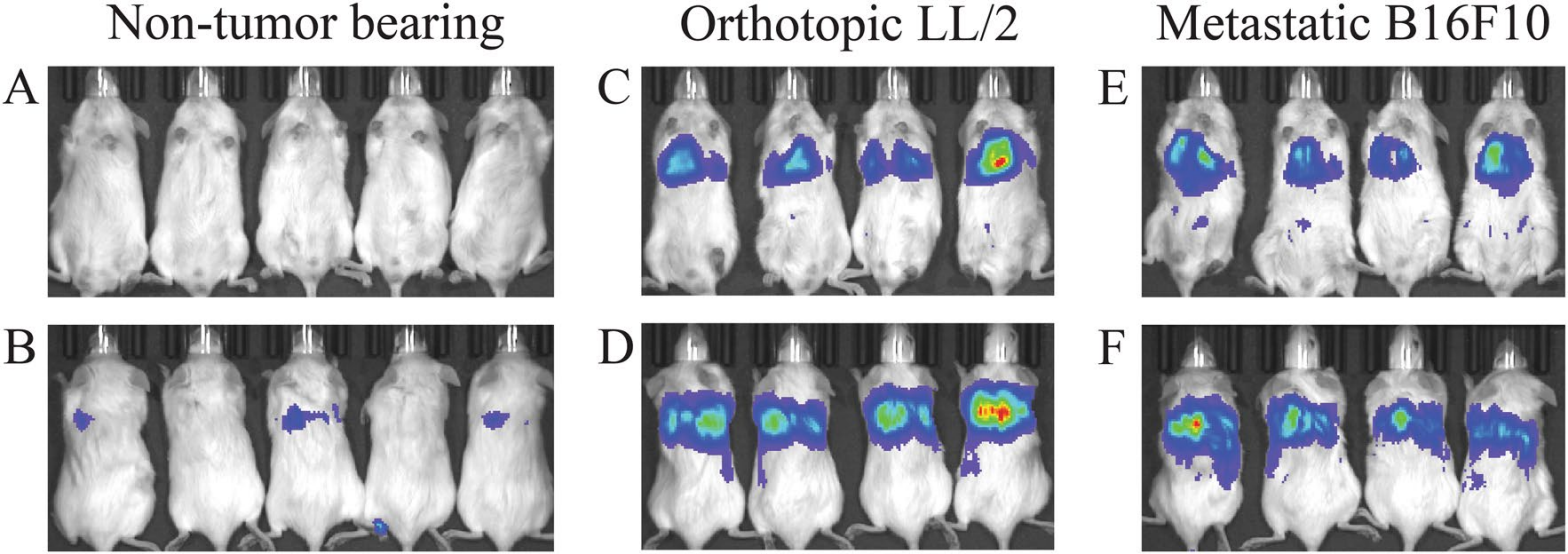
Bioluminescence images of NSG mice (LL/2 or B16F10) intravenously injected with PEG-fluc FNC particle. At 7 d post injection of the LL/2 tumor cells or 14 d post injection of B16F10 tumor cells, mice were injected with reconstituted FNC particles expressing firefly luciferase. Mice were imaged 48 h post injection of the particles with IVIS Spectrum In Vivo Imaging System. Ventral (**A**, **C**, **E**) and dorsal (**B**, **D**, **F**) views of the same mice are shown for the non-tumor bearing mice (**A**, **B**), LL/2 (**C**, **D**), and B16F19 (**E**, **F**) mice.

**Figure 3:**
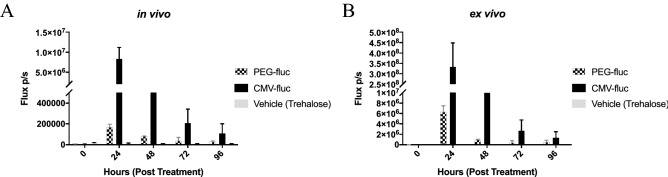
In vivo kinetics of luciferase expression from *PEG-3*-fluc plasmid formulated nanoparticles. Luciferase expression from PEG-3-fluc nanoparticles (chequered columns) was followed by bioluminescence imaging (at injection, 0 h; and at 24 h intervals up to 96 h) and compared to CMV-fluc (positive) (filled columns) and vehicle (negative) controls in mice inoculated with B16F10 tumor cells (**A**). At termination, bioluminescence in the resected lungs was measured ex vivo (**B**). Both in vivo and ex vivo imaging of the treated mice showed detectable expression of luciferase in both the PEG-fluc and CMV treated mice by 24 h, which continued for at least 96 h.

The preference for human cancer cells of the *PEG-3* promoter has been previously demonstrated in vitro and in vivo^26,27^. To test the activity of the *PEG-3* promoter in our in vivo models, LL/2 or B16F10 cells were intravenously injected via the tail vein to establish tumors in the lungs of NSG (NOD-SCID IL2rγ^null^) mice. Once the tumors had established, a single dose of nanoparticles, containing a CpG-free variant of firefly luciferase (fluc) under the control of either *PEG-3* or the CMV promoter, were injected via the tail vein at a dose of 40 μg DNA per animal. Expression was monitored and confirmed 48 h post injection of the nanoparticles (Fig. 2) and compared with healthy controls. In a second study, the in vivo kinetics of luciferase expression from the nanoparticles formulated with *PEG-3*-fluc plasmid was followed by bioluminescence (at injection and 24 h intervals up to 96 h) and compared to CMV-fluc and vehicle controls in B16F10 infected mice. The in vivo imaging of the treated mice showed detectable expression of luciferase in both the PEG-fluc and CMV-fluc treated mice by 24 h, which continued for at least 96 h (Fig. 3). As expected from in vitro data, the mean signal from the constitutively active CMV promoter was considerably higher, approximately 50-fold, than that of *PEG-3* at 24 h but the difference had diminished to 15-fold at 48 h, and was only fourfold by 96 h. It is likely that the loss of activity over time is due to loss of the plasmid from the cells, and the accelerated loss of activity of CMV activity compared to the *PEG-3* promoter is due to the known susceptibility of the promoter to methylation^28^. Ex vivo bioluminescent analysis of the lungs confirmed the in vivo data. Regression lines could not be fitted unambiguously to the CMV data, but the half-life of the luciferase expression from the PEG-3 promoter using a one phase decay least squares fit for nanoparticles (N/P = 6) was 19 h. These data indicated that a timeline of repeat dosing of between 3 and 4 days would be suitable for the following efficacy studies.

### Survival was extended with mIL-12 and TK-hIL2-mIL12 in a LL/2 orthotopic mouse model of primary lung cancer

We employed LL/2 orthotopic model in C57BL/6 mice to evaluate efficacy of the nanoparticles in the target organ (Fig. S8, A–C). Animals were euthanized when body weights had declined below 15% or a clinical condition was reached; in practice, this occurred between Study Days 12 and 23 (with a single exception in the PEG-lucia group). In parallel, an additional three mice per treatment group were used as satellites to study the toxicity of the nanoparticles on Day 9 (i.e. 1 day after the second dose). No abnormal readings were observed for blood markers for liver toxicity, among other indicators, compared to control mice, demonstrating that the treatment was well tolerated (Table S2). The study was terminated at Day 23 when only six animals remained alive (one treated with PEG3-TK-hIL2-mIL12 plasmid formulated nanoparticles and five treated with PEG3-mIL12 plasmid formulated nanoparticles).

**Figure 4:**
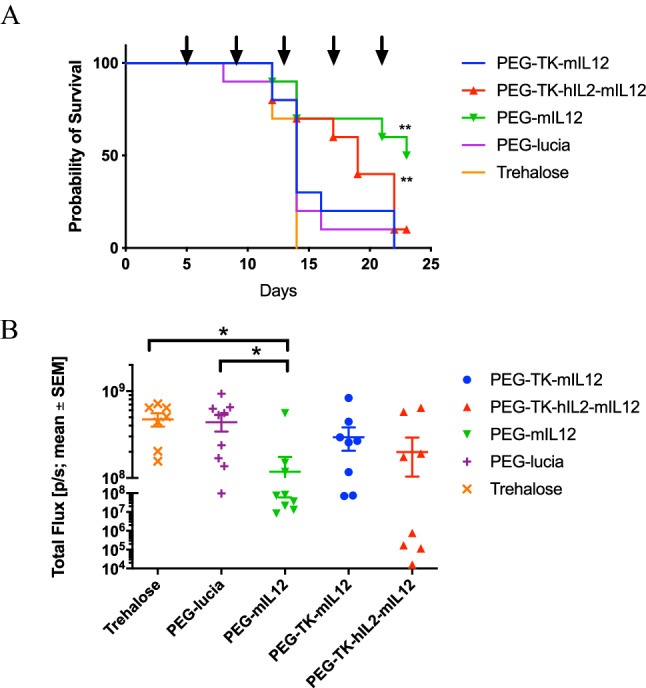
Activity of l-PEI/DNA nanoparticles in an LL/2 syngeneic, orthotopic model of primary lung cancer in C57BL/6 mice represented by a Kaplan Meier survival plot. Mice were inoculated orthotopically with LL/2 Red-FLuc murine lung cancer cell line and treated with nanoparticles at 4 d intervals, beginning at Day 5 (post tumor cell inoculation), as indicated by the arrows above the chart. The study was terminated on Day 23. Both PEG-TK-hIL2-mIL12 and PEG-mIL12 nanoparticles (red triangle and green upturned triangle, respectively) significantly (*p* ≤ 0.001, Log-rank test) extended survival in this model compared to the vehicle control (9.5% trehalose control, solid line, open circle) and PEG-lucia (nanoparticle control, solid line, filled diamond (**A**). Anti-tumour activity of PEG-3 nanoparticles was assessed through comparison of the mean in vivo luminescence signal ± SEM (Total Flux (p/s)) in the lungs of mice at Day 13 after implantation of LL/2 Red-FLuc cells orthotopically into the lungs of C57BL/6 mice. There was a significant reduction in signal (*p* ≤ 0.05, Dunnett’s multiple comparisons test) in the PEG-mIL12 group compared to the Trehalose vehicle control group indicating significant inhibition of tumor growth (**B**).

Survival time was significantly (*p* ≤ 0.05, Log-rank test) prolonged in animals receiving nanoparticles containing PEG3-TK-hIL2-mIL12 or PEG3-mIL12 plasmids compared with vehicle (9.5% Trehalose) control. The median survival times from Kaplan Meier analysis (Fig. 4A) were 19 days, 23 days and 14 days, respectively. However, median survival times for animals treated with nanoparticles formulated with PEG3-TK-mIL12 plasmid and PEG-lucia CpG-free control plasmid was 14 days, the same as the 9.5% Trehalose vehicle control. The lack of efficacy of the PEG3-TK-mIL12 plasmid was believed to be related to the poor delivery of this plasmid in vivo compared to PEG3-mIL12. Quantitative assessment by qPCR of nanoparticle transfection of the tumors in the 3 satellite mice per group 1 day after the second dose (at Day 9) demonstrated that all the plasmids had transfected the tumor cells (Fig. S5A). However, the HSV1-TK carrying plasmids appeared to have lower transfection efficiencies than either the control PEG-lucia or PEG-mIL12 possibly correlating with the larger sizes of these plasmids (Fig. S2). Interestingly, although expression of IL-12 from the PEG-TK-mIL12 nanoparticles in the tumors was higher than from PEG-TK-hIL2-mIL12, this did not translate into improved survival, but the numbers of animals tested were too small to show significance in this satellite group (Fig. S5B). In vivo imaging of luminescence, indicative of growth of LL/2-Red-Fluc cells, at Day 13 (i.e. 4 days after the second dose of nanoparticles) showed a significantly lower signal (*p* ≤ 0.05, Dunnett’s multiple comparison test) in mice treated with PEG-mIL12 nanoparticles compared with the vehicle control group or the nanoparticle control PEG-lucia group, indicating reduced growth of lung tumors in the IL-12 treatment group (Fig. 4B). In a second study of the LL/2 efficacy model (continued until all animals had reached an ethical endpoint rather than per protocol termination at Day 23), mice in the PEG-mIL12 group survived up to 25 days (median survival 21 days compared to 13.5 days for the vehicle control) but in this study the PEG-TK-mIL12 group also had an improved median survival of 20 days (Fig. S6).

CD45^+^ lymphocyte populations were significantly reduced (at termination) in the tumors of LL/2 tumor bearing mice treated with PEG-mIL12 compared with PEG-lucia and vehicle controls, but there was no significant difference in the levels of CD4^+^CD8^−^ or CD8^+^CD4^−^ cells between the three groups (Fig. S7). Populations of CD4^+^FoxP3^+^CD25^+^ suppressor T*regs* were reduced compared to the control groups, but not significantly. Therefore, no strong correlation could be made between efficacy and T cell populations.

### Survival was extended with mIL-12 and mIL2-mIL12 nanoparticles in a B16F10 experimental metastasis mouse model of lung cancer

We tested efficacy in treating multiple metastatic lesions in the lung in an experimental ‘metastatic’ tumor model of B16F10-Red-Fluc melanoma cells (Fig. S8, D–F). While the aim of the model was primarily to establish disseminated tumors in the lung, the B16F10 cells also colonized other organs as indicated by masses present on either the dorsal thoracic region, snout, neck, limb, head, tail or abdomen of individual animals (Table S3). We injected nanoparticle via the tail vein every 3 days, as the previous 4-day treatment schedules were well tolerated in the LL/2 model. Despite the nanoparticles being well tolerated in the LL/2 model, there was a decline in body weight at Day 6 (1 day after the first dose of nanoparticles) in the B16F10 model, which led to a decision to reduce the dose from 2 mg DNA/kg to 1 mg/kg for the second dose for all groups at Day 8 as a precautionary measure. All the mice recovered sufficiently to receive the per protocol treatment for subsequent doses at Study Days 11, 14 and 17. All animals evaluated showed micro-metastases in lung sections, but lung surface macro-metastases were not quantifiable in the majority of animals (in all groups) due to the high number of metastases resulting in overlapping lesions (Table S3).

**Figure 5:**
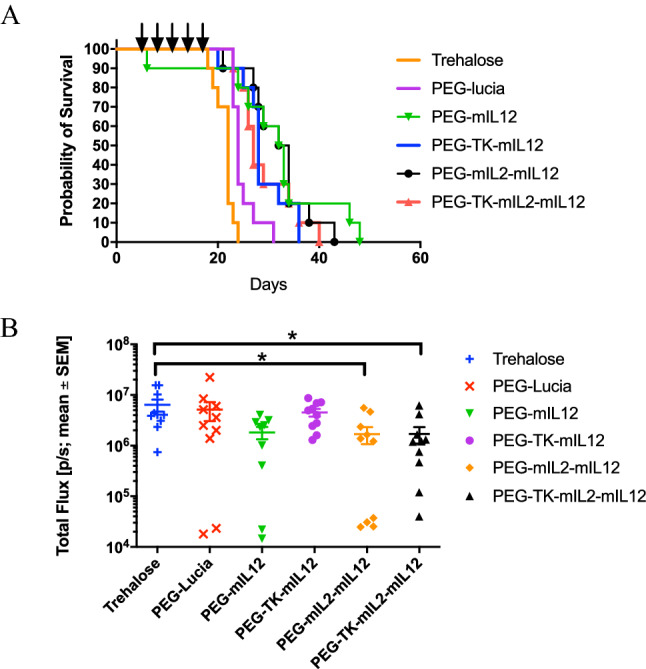
Activity of l-PEI/DNA nanoparticles in a B16F10 syngeneic, metastatic model of cancer in the lungs in C57BL/6 mice represented by a Kaplan Meier survival plot. Mice were inoculated intravenously via the tail vein with B16F10-Red-FLuc murine melanoma cells and treated with nanoparticles at 3-d intervals, beginning at Day 5 (post tumor cell inoculation) as indicated by the arrows above the plot. Nanoparticle formulations of plasmids PEG-mIL12 (green line, filled upturned triangle), PEG-TK-mIL12, PEG-mIL2-mIL12, PEG-TK-mIL2-mIL12 significantly extended survival (*p* ≤ 0.01, Log-rank test) of the mice compared to vehicle control and PEG-lucia (*p* ≤ 0.01, Log-rank test) (**A**). PEG-lucia also significantly extended survival in this study compared to the vehicle control (*p* ≤ 0.05, Log-rank test). Plot of the mean in vivo luminescence signal ± SEM (Total Flux (p/s)) in the lungs of C57BL/6 mice at 12 d after inoculation of B16F10-Red-FLuc cells showed a significant reduction in signal between PEG-mIL2-mIL12 and the vehicle control group, and PEG-TK-mIL2-mIL12 and the vehicle control group (*p* ≤ 0.05, Dunnett’s multiple comparisons test) (**B**).

Median survival times for all treatment groups were significantly (*p* ≤ 0.01, Log-rank test) improved compared to the 9.5% trehalose control (9.5% trehalose, 22 d; PEG-mIL12, 32.5 d; PEG-TK-mIL12, 28 d; PEG-mIL2-mIL12, 33 d; PEG-TK-mIL2-mIL12, 27 d). The PEG-lucia control also improved survival compared to the vehicle control (24 d vs. 22 d, respectively), however, there was also a significant (*p* ≤ 0.05, Log-rank test) difference between the nanoparticles that expressed cytokines and the PEG-lucia control, demonstrating that improved survival was due to the expression and action of the cytokine rather than stimulation of innate immunity from the polymer or the plasmid (Fig. 5A). Luminescence readings in the lungs on Study Day 12 were significantly (*p* ≤ 0.05, Dunnett’s multiple comparison test) lower in the group treated with mIL2-mIL12 and the TK-mIL2-mIL12 groups compared with the vehicle control and there was a trend towards significance for the group of mice treated with PEG-mIL12 (*p* ≤ 0.054, Dunnett’s multiple comparison test) (Fig. 5B).

### Nanoparticle delivery of mIL-12 was more effective than recombinant mIL2 in a B16F10 experimental metastasis mouse model of lung cancer

**Figure 6:**
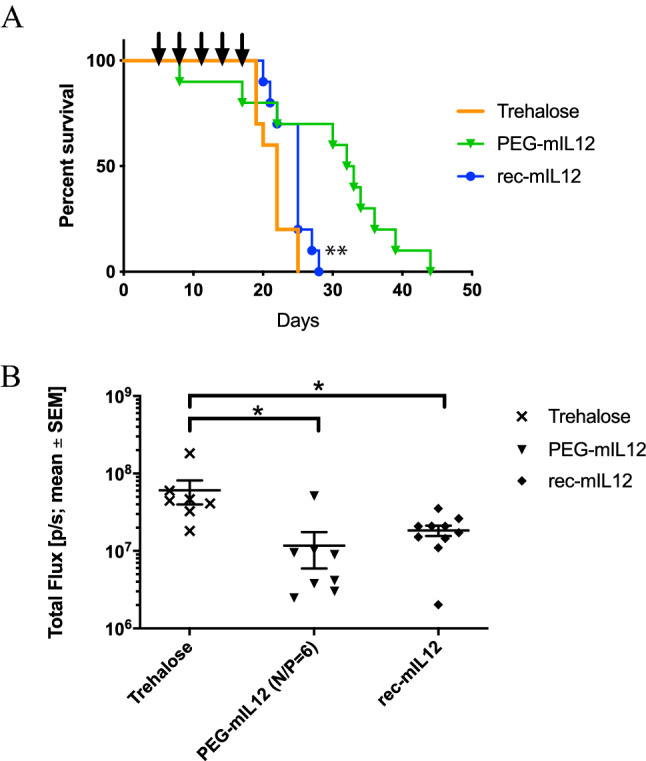
Comparison of l-PEI/PEG-mIL12 plasmid DNA nanoparticles with recombinant murine IL-12 protein in a B16F10 Red-FLuc syngeneic, metastatic model of cancer in the lungs of mice using Kaplan Meier survival analysis. (**A**) Nanoparticles were dosed at 3-d intervals, beginning on Day 5 (post tumor cell inoculation) as indicated by the arrows above the plot. The nanoparticle formulations produced a significant survival benefit over vehicle control (9.5% trehalose) (*p* ≤ 0.01, Log-rank test) and over the recombinant murine IL-12 (*p* ≤ 0.05, Log-rank test) at the dose tested. (**B**) Comparison of the mean in vivo luminescence signal ± SEM (Total Flux (p/s)) in the lungs of C57BL/6 mice 19 days after inoculation of B16F10-Red-FLuc cells showed a significant reduction in signal between PEG-mIL12 nanoparticles (upturned solid triangle) and the vehicle control group and also between recombinant mIL-12 (filled circle) and the vehicle control group (*p* ≤ 0.05, Dunnett’s multiple comparisons test).

In a following study of mice bearing B16F10 tumors, the anti-tumor effect of nanoparticles expressing mIL-12 was compared to recombinant mIL-12 protein administered subcutaneously. The dose of recombinant IL-12 protein was chosen so that the toxic side-effects of recombinant IL-12 would be minimized in this study, yet the protein would still be therapeutically effective^29,30^. Nanoparticles were dosed as described in the previous section. For the recombinant protein, the animals were treated initially with a sentinel dose of recombinant murine IL-12 at 4 μg/kg at Day 5 followed by four subsequent doses of 12 μg/kg at the same intervals as the nanoparticles (Days 8, 11, 14 and 17). Median survival times were significantly longer for animals treated with PEG-mIL12 nanoparticles (32.5 d, *p* ≤ 0.01, Log-rank test) and recombinant mIL-12 (25 d, *p* ≤ 0.05, Log-rank test) than those with the trehalose control (22 d). Additionally, the median survival time following treatment with PEG-mIL12 nanoparticles was significantly (*p* ≤ 0.05, Log-rank test) longer than recombinant mIL-12 at the dose tested (Fig. 6A). Luminescence readings on Study Day 19 indicated significant (*p* ≤ 0.05, Dunnett’s multiple comparison test) inhibition of tumor growth by treatment with PEG-mIL2 and rec-mIL12 compared with 9.5% trehalose control (Fig. 6B).

## Discussion

Payload choice, the nature of the disease, and the limitations of the delivery system are important considerations in the design of therapeutic nanoparticles. For cancer therapy, we believed that repeated administration would be necessary to provide a safe, lasting, and effective anti-tumor response as the proportion of tumor cells transfected by each dose was likely not to be 100%. We have adopted an approach that uses a non-viral nanoparticle to deliver a plasmid comprising a promoter that is preferentially active in tumor cells and a therapeutic cytokine (IL-12) molecule. Synthetic gene delivery systems are known to be inefficient, yet can be used for repeated administration, whereas viral delivery vehicles can be more efficient but are antigenic^11,31^. In addition to the selectivity conferred by the *PEG-3* promoter (discussed below), tumor selectivity might also arise through the EPR effect of nanoparticles^13^. Our nanoparticles, sized 130 nm, are below the size range of functional pores within tumor blood vessels (380–720 nm) and hence are expected to benefit from the EPR effect. Our l-PEI/DNA nanoparticles remain untargeted to any cell-surface receptor where issues of exclusive expression of the antigen on the tumor or escape mechanisms might prevail^12^. Systemic delivery would allow access to disseminated metastases within the lung but this route of administration can be hampered by clearance by the mononuclear phagocytic system (MPS) and reticuloendothelial system (RES) which can, ultimately, contribute to toxicity as high local concentrations accumulate in the Kupffer cells in the liver^12^. In our studies, the l-PEI/DNA nanoparticles were manufactured by the FNC method, which produces consistent batches of nanoparticles with controllable polymer and payload contents appeared to alleviate any permanent systemic toxicity^16^.

As previously reported, the *PEG-3* promoter is preferentially active in tumor cells, requiring key transcription factors such as such as AP-1 and PEA-3, thus providing cancer selectivity^26^. This is demonstrated in our experiments using a luciferase gene driven by the *PEG-3* promoter in healthy mice, or mice injected with LL/2 (Lewis lung carcinoma) or B16F10 tumours, where expression is clearly seen in the lungs of the diseased mice compared with those of healthy mice.

In our product design, we also aimed to limit off-target cytokine release due to inappropriate activation of Toll-like receptors (TLRs); so we reduced CpG sequences within the plasmid DNA as far as is practicable. In the final plasmid, only the wild-type *PEG-3* promoter sequence contains CpG sequences, as attempts to reduce these either altered the strength of the promoter or its selectivity. Reducing CpG content is practiced in gene therapy to limit inflammation and inactivation of gene expression through methylation, but it is not an obvious choice in the field of cancer treatments as CpG sequences have previously been used as adjuvants to cancer vaccines to stimulate the innate immune response through TLR9 activation^32^. Thus, the CpG-reduced plasmid nanoparticle showed lower levels of inflammation markers compared to nanoparticles with the CpG-rich content. The limited immune activation observed with the formulated CpG-reduced plasmid nanoparticle might have been caused by the remaining 43 CpG sites within the wild-type *PEG-3* promoter or possibly by the PEI polymer itself, as this can stimulate TLR4 signalling. Furthermore, it was important for us to demonstrate that efficacy in the tumour models was dependent upon the expressed gene product, and not stimulation through activation of TLR9 innate immune responses by CpG sites, as a previously published by Rodrigo-Garzón et al. of a l-PEI/plasmid nanoparticle expressing IL-12 in a Lewis lung carcinoma model^33^. It is clear from our results in both in the LL/2 and B16F10 models that efficacy is driven by IL-12 protein expression rather than the DNA content per se.

As stated above, we believed that repeated administration would be necessary to provide a safe, lasting, and effective anti-tumor response. Therefore, we sought a payload that had a considerable anti-tumour bystander effect such as IL-12, which has been used as a systemic recombinant protein treatment in the clinic but has been withdrawn due to a poor safety profile at high concentrations. However, IL-12 does have desirable anti-cancer properties, being able to stimulate both the innate and adaptive arms of the immune system^34^. We hypothesized that efficacy of IL-12 could be maintained and safety improved if expression was confined to the tumour site resulting in high local concentrations where it would have the greatest effect and minimal concentrations elsewhere where undesired toxicity might occur. For our efficacy studies in vivo, we chose models, either orthotopic or metastatic, where the tumours developed in the lungs of immune competent syngeneic mice to reflect more faithfully the biology of lung tumour growth and immune stimulation^35^. Two cell lines were chosen that are known to be intractable to immunotherapy, B16F10 and LL/2^36,37^. The LL/2 orthotopic model represented a primary tumour model and intravenous administration of B16F10 cells modelled metastatic disease in the lung. Schedules of repeat dosing of every 3 or 4 days were undertaken in the B16F10 and LL/2 models, respectively, based upon preliminary wash-out experiments. After the first injection, the animals suffered weight loss in all the groups receiving the nanoparticles in both the LL/2 and the B16F10 models, but this effect appeared to be temporary and related to polymer administration rather than to the expressed gene as the same effect was noticeable in the control plasmid-nanoparticle group (PEG-lucia). Markers of liver toxicity were not elevated after two nanoparticle doses in the LL/2 model, indicating that this was not related to liver damage. In the LL/2 tumor model, the IL-12 only expression plasmid (PEG-mIL12) resulted in the biggest overall survival benefit. In vivo bioluminescence at Day 12 also suggested that this plasmid had the greatest anti-tumour effect (lowest tumour-driven bioluminescent signal in the lung) in support of the survival data. The expression cassette containing HSV1-TK-hIL2-mIL12 (for LL/2) or HSV1-TK-mIL2-mIL12 (for B16F10) also significantly improved the survival of the mice in these models, demonstrating that these three gene expression cassette could also produce therapeutically relevant amounts of either IL-2 or IL-12 or both. Thymidine kinase was not used in a therapeutic capacity in this model although a previous report had shown therapeutic responses with 5-fluorocytosine and valganciclovir treatment when administered in combination with cytosine deaminase, HSV-TK and IL-12 in a TRAMP-C2 prostate adenocarcinoma model^38^. In our B16F10 study, the plasmid expressing the single cytokine IL-12 (under the control of the *PEG-3* promoter) again demonstrated the best survival benefit over plasmid expressing both IL-2 and IL-12. IL-2 and IL-12 cytokines reciprocally upregulate each other’s receptors and use separate signalling pathways to induce different but complementary biologic effects^39^. The combination of IL-2 and IL-12 was shown to be complementary in creating a greater anti-tumor response than the individual cytokines against neuro 2A tumours^40^, however, in our study the therapeutic effect was greatest with IL-12 alone. The efficacy of the PEG-mIL12 nanoparticle was the compared with recombinant murine IL-12 protein administered subcutaneously. The nanoparticle formulations extended survival beyond that achieved by the systemically administered recombinant protein, thus demonstrating efficacy at least equivalent to the recombinant protein. This indicates that expression of IL-12 in tumor cells can induce equivalent efficacy to the systemically administered recombinant protein.

Systemic IL-12 monotherapy has failed to deliver on the promise of early animal data, although, complete responses have been noted in AIDS-related Kaposi’s sarcoma and cutaneous T-cell lymphoma, plus additional minor responses in non-Hodgkin lymphomas (NHLs), renal cell carcinoma (RCC) and melanoma^20,34^. Recent studies to locate IL-12 to the tmor microenvironment via anchoring with collagen showed enhanced therapeutic index in preclinical models suggesting the feasibility of targeted IL-12 therapy^41,42^. Local delivery of plasmid DNA using electrogene therapy has provided efficacy in dogs, indicating that expression of IL-12 in tumours could still be a viable therapeutic approach^43–46^. Therefore, the potential to delivery systemically a safe vehicle to express IL-12 within the tumor microenvironment at suitable levels for efficacy offers new alternatives for the therapeutic use of IL-12. We believe that the delivery of the nanoparticle formulations described in our study, which are specifically active in the tumour microenvironment, can offer new alternatives for IL-12-based therapy as monotherapy and in combination with checkpoint inhibitors.

## Materials and methods

All animal experiments and procedures were approved by Institutional Animal Care and Use Committees of the Johns Hopkins University and performed in compliance with the Animal Welfare Act regulations and Public Health Service (PHS) Policy. Johns Hopkins University has an approved PHS Assurance. All animal experiments presented in the manuscript followed the recommendations in the ARRIVE guidelines.

### Plasmids

Payload coding sequences were optimized to replace CpG sites and rare codons, according to human codon usage database^47^. The coding regions of the payloads were modified to remove CpG sequences that might provoke an adverse immune response through Toll-like receptor 9 (TLR9) activation in vivo^48^. In some plasmids the HSV1-TK gene was inserted under the control of the *PEG-3* promoter as a potential imaging and therapeutic gene and in other constructs an IL-2 gene was inserted to potentially synergise with IL-12. These additional coding regions were separated by 2A ribosome skipping sequences to allow the discrete expression of the individual proteins from the *PEG-3* promoter^49,50^. The termini of the sequences were modified to include a 5′ restriction enzyme site compatible with the pCpG free-N-mcs plasmid (Invivogen, San Diego, CA), in which the *PEG-3* promoter was cloned in place of the mCMV enhancer and EF1 promoter, and a stop codon followed by an NheI site at the 3′ end. Cytokines were cloned in isolation or in combination with additional reporter gene payloads such as CpG-free HSV-1 TK (TK) or CpG-free firefly luciferase (fluc). These cytokines include: murine single chain IL-12 (mIL12)^23^; murine IL-2 (NCBI NM_008366.3) plus murine IL-12 (mIL2-mIL12); and human IL-2 (Genbank S77834.1) plus murine IL-12 (hIL2-mIL12). Where there were two open reading frames (ORFs) in the cassette, the first ORF was followed by a 2A ribosome skipping sequence in frame with the gene sequence. Where three genes were cloned, a furin cleavage site (RRKR) and GSG linker and a 2A site were used in between the second and third open reading frames (Fig. S2)^49^. Native leader sequences were used for IL-2 and IL-12 genes.

### In vitro testing

The *PEG-3* promoter activity was tested by transfection of a PEG-luciferase plasmid into cultured cancer cells, such as human lung cancer cell lines H460 (ATCC® HTB-177), H1299 (ATCC CRL-5803), EMT6 (ATCC CRL-2755), murine lung cancer cell line LL/2 (ATCC CRL-1642), or murine melanoma cell line B16F10 (ATCC CRL-6475). All tests included a transfection control plasmid containing *Renilla* luciferase under the control of an SV40 promoter and the luciferase activity was assayed according to manufacturer’s instructions using the Dual-Luciferase Reporter Assay System (Promega, Madison MI). For assays to determine cytokine expression, constructs were transfected into cultured LL/2-Red-Fluc (Perkin Elmer, Watham, MA). Plasmids were formulated with PEI*pro* (Polyplus Transfection, Illkirch, France) according to the manufacturer’s instructions. For example, LL/2 cells were plated at a density of 1 × 10^5^ cells/3.8 cm^2^ in a 12 well plate in DMEM. 1 µg of plasmid was diluted into 25 µL of serum free media and vortexed gently. 4 µL PEI*pro* was added into 25 µL of serum free media and the PEI*pro* solution was added to the DNA solution and vortexed gently, followed by 15 min incubation at room temperature. The cells were incubated at 37 °C in 5% CO_2_ for 48 h. Culture supernatant was then removed and stored at − 20 °C until tested by ELISA using the relevant anti-cytokine Quantikine ELISA kit (R&D Systems, Minneapolis, MN) according to the manufacturer’s instructions. Dilutions of the culture supernatants were made in duplicate and quantitation of cytokine expression was measured against standard curves of known standards.

### Thymidine kinase activity assay

1.5 µg of PEG-TK-hIL2-mIL12, PEG-TK-mIL12, PEG-mIL12 or PEG-lucia plasmid was diluted in 75 µL of pre-warmed OptiMEM medium and gently vortexed. 12 µL of PEIpro reagent was diluted into 75 µL of OptiMEM. The PEIpro solution was then added to the DNA solution and vortexed gently. The DNA/PEIpro solution was incubated for 15 min at room temperature. 2.5 µL of the DNA/PEIpro solution was added to the 96-well plate. Each well was plated with 5000 cells. LL/2-Red-FLuc cells (Perkin Elmer, Waltham, MA) were cultured in a T175 flask until 60–70% confluent. The cell monolayer was briefly washed with 20 mL PBS, trypsinized with 3 mL of trypsin/EDTA for 3 min, and 7 mL of media was added once the cells were removed from the surface. The suspension was transferred to a 15-mL Falcon tube and centrifuged at 200 × *g* for 5 min. The supernatant was removed, and the cell pellet was resuspended in 3 mL of fresh media. Cells were plated at 1000 or 5000 (assay dependent) cells/well in a 96-well plate in 100 µL per well of complete DMEM media. Plates were transferred to a 37 °C/5% CO_2_ incubator and allowed to grow for 24 h prior to compound treatment. A 100 mM stock was prepared in DMSO and used to prepare a tenfold dilution series from 1000 mM to 0.01 µM in DMSO. The media containing transfection reagent were removed from the transfection plate and replaced with 50 µL/well of respective ganciclovir (Cytovene, Roche, Montreal, Quebec, Canada) concentration (triplicate wells for each concentration). The plate was incubated for 48 h at 37 °C. CellTox Green Cytotoxicity reagent (Promega, Madison, WI) was made up to 2× with assay buffer and 50 µL of reagent was added to each well of the 96-well plate containing the cells incubated with ganciclovir. The plate was protected from light, incubated for 15 min at room temperature and the green fluorescence was read at 485 nm (excitation) and 520 nm (emission).

### Functional analysis of expressed IL-2 and mIL-12- CTLL-2 assay

An assay based upon that of Weston et al.^51^ was set up to measure functional IL-2 and IL-12^52^. The CTLL-2 cell line is a cytotoxic T cell line of murine origin derived from C57BL/6 inbred mice (H-2b) and is dependent upon stimulation from IL-2 for survival and growth^51^. Therefore, the activity of the expressed proteins in this assay was of direct relevance to the efficacy in syngeneic tumor models in C57BL/6 mice. CTLL-2 cells (ECACC 93042610), maintained at 2 × 10^5^ cells/mL in complete RPMI media (containing T-Stim, Corning, Flintshire, UK), were collected and centrifuged at 400 × *g* for 5 min. Cells were re-suspended in 20 mL of RPMI media containing all additional supplements except T-Stim and cultured further for 24 h at 37 °C in 5% CO_2_. Cells were then plated at 4 × 10^4^ cells/well in a 96-well plate in 50 µL of RPMI media (without T-Stim) on the final cell proliferation plate. In order to assay proliferation, 100 µL of CellTiter-Glo Reagent (Luminescent Cell Viability Assay, Promega Corp., Madison, WI) was added to the cells in line with the manufacturer’s guidelines for the reagent. Cells were incubated at room temperature with shaking at 500 rpm for 15 min and the luminescence was recorded on a luminometer and quantified using a standard curve as per manufacturer’s instructions. As a positive control, lyophilized recombinant hIL-2 (rhIL-2) was reconstituted to 100 µg/mL in 100 mM sterile acetic acid containing 0.1% BSA. Stock rhIL-2 was diluted down to 500 ng/mL in RPMI 1640 (without T-Stim), and this was used to prepare a twofold dilution series from 20 to 0.163 ng/mL in a 96-well intermediate plate in a final volume of 100 µL/well. 50 µL of each dilution was transferred into the final cell proliferation plate. A twofold dilution series from 1:2 to 1:32 for cell culture supernatants from plasmid transfections was prepared in RPMI 1640 (without T-Stim) (125 µL:125 µL media). 50 µL of each dilution was then transferred into the final cell proliferation plate.

### Functional analysis of expressed IL-12 in vitro in a PBMC stimulation assay

Peripheral blood mononuclear cells (PBMCs) were isolated from whole blood samples from heathy donors by Ficoll Hypaque gradient centrifugation. 1 × 10^7^ PBMCs were added to a total of 20 mL supplemented medium (RMPI (Thermo Fisher, Loughborough, UK), 10% heat inactivated foetal bovine serum (Sigma Aldrich, Dorset, UK), 2 mM L-glutamine (Sigma Aldrich, Dorset, UK), 1% 100× penicillin streptomycin solution (Sigma Aldrich, Dorset, UK)) in a 75 cm^2^ culture flask. 20 µL of 10 mg/mL phytohemagglutinin (PHA) (200 µg PHA) was added and the flask was incubated for 3 d at 37 °C in 5% CO_2_. 20 mL of supplemented media was added and then gently mixed by shaking. 20 mL of the contents were then transferred to a clean 75 cm^2^ culture flask and human recombinant IL-2 was added to 50 U/mL and further incubated for 24 h at 37 °C in 5% CO_2_. PBMCs were diluted to 2 × 10^5^ cells/mL for use in the assay.

A 96-well plate was coated with 5 µg/mL mouse anti-IL-12 antibody in NaCO_3_ or PBS buffer and incubated at 4 °C overnight. Plates were washed with buffer and then blocked with 1% BSA/PBS for 1 h at room temperature. Serial dilutions of mIL-12 reference compound (5 to 0.008 ng/mL) and cell supernatant (containing expressed mIL-12) were made and 100 µL of reference or test sample dilutions were added to the wells, followed by incubation for 2.5 to 3 h at room temperature. The plate was washed with PBS buffer and 100 µL PHA stimulated PBMC cells were added (2 × 10^4^ cells/well). The cells were incubated for 7 d at 37 °C in 5% CO_2_. Cell proliferation was detected using CellTiter-Glo Reagent according to the manufacturer’s instructions.

### Nanoparticle formulation with FNC process

For in vivo experiments, nanoparticles comprised of the plasmid and l-PEI (in vivo-jetPEI, Polyplus Transfection, Illkirch, France) were prepared using the FNC process on a confined impinged jet (CIJ) device^14,16^. The CIJ device and all the fittings were autoclaved on a dry cycle prior to use. A working solution of in vivo-jetPEI was made in 9.5% trehalose and combined under pressure with a stock solution of plasmid in 9.5% trehalose. *PEG-3* plasmids containing CpG-free genes for mIL-12, TK-mIL-12, TK-hIL2-mIL12, TK-mIL2-mIL12, mIL2-mIL12 (PEG-mIL-12, PEG-TK-mIL12, PEG-TK-hIL2-mIL12, PEG-TK-mIL12-mIL12, PEG-mIL2-mIL12, respectively) or lucia luciferase (Invivogen, San Diego, CA) (PEG-lucia) were formulated at a N/P ratio of 6 and a final DNA concentration of 200 µg/mL, and lyophilized.

### Nanoparticle formulation with FNC process

Nanoparticles comprised of the payload plasmid, including PEG-3 plasmids containing CpG free genes for mIL-12, TK-mIL-12, TK-hIL2-mIL12, TK-mIL2-mIL12, and mIL2-mIL12 (PEG-mIL-12, PEG TK-mIL12, PEG-TK-hIL2-mIL12, PEG-TK-mIL12-mIL12, and PEG-mIL2-mIL12, respectively) or lucia luciferase (Invivogen, San Diego, CA) (PEG-lucia), and lPEI (in vivo-jetPEI, Polyplus Transfection, France) were prepared in a reproducible and scalable manner with controlled nanoparticle size by flash nanocomplexation (FNC) method, as detailed in our previous reports^23^. Briefly, the plasmid (at a concentration of 400 μg/mL) and lPEI (at an N/P ratio of 6) were dissolved separately in 9.5% trehalose solutions, with pH of lPEI solutions adjusted to 3.5. After sterilization of all parts by autoclave, the solutions were loaded onto syringes and connected to the inlets of a confined impinging jet (CIJ) device via PTFE tubing. Controlled by a digital syringe pump, the solutions were then steadily injected into the CIJ device at a flow rate of 20 mL/min. The turbulent mixing in the CIJ device allowed a superfast mixing of the materials, thus enabling controlled nanoparticle assembly kinetics that resulted in a controlled nanoparticle size and composition^14^. The production is an operator-independent process^16^ that generated large quantities of nanoparticles with the same properties across different batches of studies. The nanoparticles were lyophilized into a shelf-stable form via drying under 10 Pa and 20 °C for 24 h, which were reconstituted into a stable suspension by addition of the original volume of sterile water before injection.

### In vivo and ex vivo imaging

4–6 week old NSG (NOD-SCID IL2rγ^null^, Johns Hopkins University Animal Animal Resource Core) were injected with 5 × 10^6^ LL/2 or B16F10 cells intravenously. 7 d (for LL/2) or 14 d (for B16/F10) post injection of the cells, mice were injected with reconstituted FNC nanoparticles. Mice were imaged at 24, 48, 72, and 96 h post injection of the particles with IVIS Spectrum In Vivo Imaging System (PerkinElmer, Waltham, MA). After in vivo imaging was completed, mice were euthanized, and the lungs were imaged ex vivo.

### In vivo cytokine release testing

6-week old CD 1 mice (Charles River, Wilmington, MA) were injected with the indicated nanoparticles via the lateral tail vein. 48 h post injection of the particles, the animals were euthanized, blood was taken, transferred to BD Vacutaner Plus SST Tube (BD Bioscience, Franklin Lakes, NJ) and serum was prepared according to the manufacturer’s protocol. The levels of murine IL-12, IFN-γ, and TNF-α in serum samples were determined by Quantikine ELISA kits (M1270, MIF00, MTA00B, R&D systems, Minneapolis, MN) according to the manufacturer’s protocols.

### In vivo efficacy LL/2-Red-Fluc mouse model

LL/2-Red-FLuc mouse lung tumour cells (Perkin Elmer, Waltham, MA, USA) were cultured in MEM supplemented with 10% FBS, 1% GlutaMAX™ and 1% penicillin–streptomycin, and grown at 37 °C with 5% CO_2_ (materials supplied by Invitrogen, Carlsbad, CA). The cells were harvested by trypsinization, washed twice in Hank’s Buffered Saline Solution (HBSS) and counted. The final cell density was adjusted with HBSS:Matrigel (BD Biosciences, East Rutherford, NJ, USA) (1:1 v/v) to 2 × 10^6^ cells/mL. Female C57BL/6 (Envigo, Indianapolis, IN) mice were inoculated while under intraperitoneally injected anaesthesia (Ketamine (14 mg/mL)/Xylazine (1.2 mg/mL)) (Clipper Distributing Company, St Joseph, MO). The skin at the injection site was liberally swabbed with alcohol and 20 µL aliquot of cell suspension containing 4 × 10^4^ LL/2-Red-FLuc cells was injected into the pleura. A 200 µL bolus dose of Buprenex (Buprenorphine-HCl, 0.01 mg/mL) (Hospira, Inc, Lake Forest, IL) was administered subcutaneously for pain relief at the time of surgery and the following day. The presence of lung tumors was confirmed based on a positive luminescence signal in the thoracic region of whole. In vivo whole-body luminescence imaging, using the Perkin Elmer IVIS®Lumina XR imaging system, was performed on all animals at inoculation (Study Day 0) and then on all remaining animals on Study Days 5, 9, 13, and at termination. Animals were administered 150 mg/kg D-luciferin (15 mg/mL solution prepared in PBS) via intraperitoneal injection and were imaged 5–10 min later while under isoflurane anaesthesia (initially 4–5% then decreased to maintenance level of 1.5–3% once the animals are anesthetized). Luminescence signal was measured in the region of interest (thoracic region) and images were captured. Images were analysed using Living Image 4.4 (Caliper Life Sciences, Hopkinton, MA). Animals (with positive luminescent signal) were randomized using a matched pair distribution method, based on body weight, into groups of 10, 5 days post-inoculation (Study Day 5). Mortality and checks for clinical signs were performed once daily in the morning during the study. Body weights were recorded for all animals on Study Day 5 and then at least twice weekly. 9.5% trehalose buffer and nanoparticles containing the plasmids mIL12, TK-mIL12, TK-hIL2-mIL12 and PEG-lucia (CpG-free plasmid control) were reconstituted to give dosing solutions of 200 µg DNA/mL. Formulated test articles were stored at 4 °C, and the time interval between reconstitution and use was minimized. Nanoparticles containing 40 µg DNA of Plasmid T183 (PEG-TK-mIL12), Plasmid P9945 (PEG-TK-hIL2-mIL12), Plasmid Q1100 (PEG-mIL12) or PEG-lucia CpG-free control, or 9.5% trehalose control; were administered via intravenous injection (i.v.) in a fixed dose of 40 µg per animal on Study Days 5, 9, 13, 17 and 21. Animals were terminated on reaching an ethical end-point of either body weight loss or defined adverse clinical observations. At termination, animals were anaesthetized for blood collection and euthanized by exsanguination via terminal cardiac bleed by approved standard procedures.

### In vivo efficacy B16F10 mouse model

B16F10-Red-FLuc mouse melanoma cells (Perkin Elmer, Waltham, MA, USA) were cultured in RPMI 1640 cell culture medium supplemented with 10% FBS, 1% GlutaMAX, and 1% penicillin–streptomycin, and grown at 37 °C with 5% CO_2_. The cells were harvested by trypsinization, washed twice in HBSS and counted. The final cell density was adjusted with HBSS to 3.5 × 10^6^ cells/mL. 100 µL of cell suspension, containing 3.5 × 10^5^ cells, was discharged into the tail vein of mice at the start of the study (Day 0). Imaging was performed, as described above, on study Day 5, when the presence of lung tumors was confirmed. Animal handling, monitoring, ethical procedures and termination were as described above.

Nanoparticles containing PEG-lucia, PEG-mIL12, PEG-TK-mIL12, PEG-mIL2-mIL12 and PEG-TK-mIL2-mIL12 were reconstituted in 300 μL nuclease-free water per vial (60 μg DNA per vial) on the day of dosing to give dosing solutions of 200 µg DNA/mL. Formulated test articles and 9.5% trehalose (vehicle control) were stored at 4 °C and used on day of reconstitution. Treatments were administered at a dose of 2 mg/kg in a dosing volume of 10 mL/kg on Study Days 5, 11, 14 and 17. Due to declining body weight in all groups apart from the vehicle control at Day 6, the dose was reduced to 1 mg/kg in 5 mL/kg for the dose administered on Study Day 8. Dosing then resumed at 2 mg/kg in 10 mL/kg on Study Day 11 and for subsequent doses.

## Supplementary information

Supplementary Informations.
