# Supplementary material for: Nanoparticle-mediated tumor cell expression of mIL-12 via systemic gene delivery treats syngeneic models of murine lung cancers
Source: Sci Rep. 2021 May 6;11:9733. doi: 10.1038/s41598-021-89124-4 (PMC8102550; doi:10.1038/s41598-021-89124-4)
Supplement: Supplementary file 1 — Supplementary Informations. [file 41598_2021_89124_MOESM1_ESM.pdf]

Title

Nanoparticle-mediated tumor cell expression of mIL-12 *via* systemic gene delivery treats syngeneic models of murine lung cancers.

Authors

H. Ahn[1], C. Carrington[2], Y. Hu[4], H. Liu[3,5], C. Ng[3], H. Nam[1,5], A. Park[1, ‡], C. Stace[2†], W. West[2], H. Mao[3,4,5], M. G. Pomper[1,5], C. G. Ullman[2,*,††], I. Minn[1,5,*]

Affiliations

[1]Russel H. Morgan Department of Radiology and Radiological Science, Division of Nuclear Medicine and Molecular Imaging, Johns Hopkins University, School of Medicine, Baltimore, MD 21205, USA

[2]Cancer Targeting Systems, 1188 Centre Street, Newton Centre, MA 02459, USA.

[3]Department of Materials Science and Engineering, Johns Hopkins University, Baltimore, MD 21218, USA

[4]Translational Tissue Engineering Center, Department of Biomedical Engineering, Johns Hopkins University, School of Medicine, Baltimore, MD 21287, USA

[5]Institute for NanoBioTechnology, Johns Hopkins University, Baltimore, MD 21218, USA

[†]Present address: Platform First, Clare Drive, Highfields Caldecote, Cambridge, United Kingdom, CB23 7GB.

‡Present address: AstraZeneca (MedImmune), One Medimmune Way, Gaithersburg, MD 20878

[††] Present address: Paratopix Ltd., Bishop's Stortford, CM23 5JD, UK

[*]Corresponding authors: chris.ullman@paratopix.com; iminn1@jhmi.edu

Supplementary Material

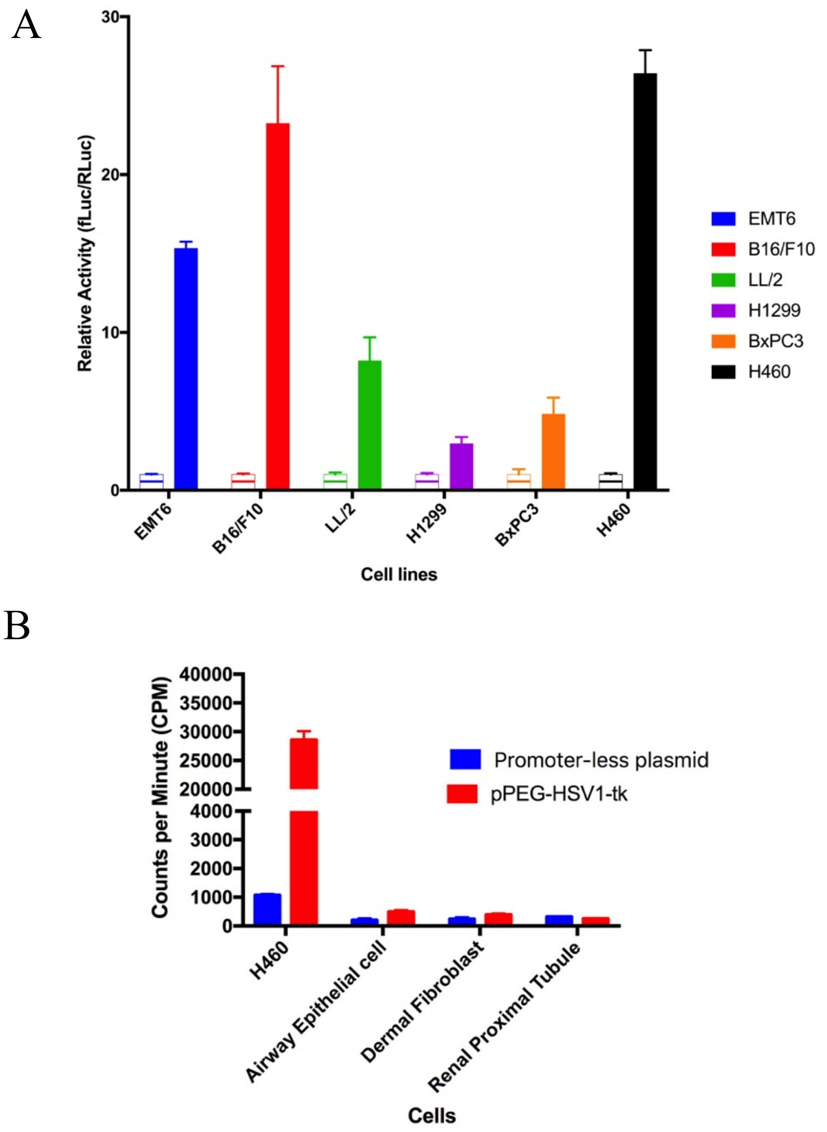

Fig. S1: The relative activities of *PEG-3* promoter in human and murine cancer cell lines and human normal cells. (A) Activity of the *PEG-3* promoter was assayed in EMT6 (murine mammary carcinoma), B16F10 (murine melanoma), LL/2 (murine Lewis lung carcinoma), H1299 (human lung carcinoma, non-small cell carcinoma), BxPC3 (human pancreas adenocarcinoma) and H460 (human large lung cell carcinoma) and compared to a plasmid control (promoter-less plasmid, dashed columns). Expression was normalized by comparing levels of firefly luciferase (fLuc) with Renilla luciferase (RLuc) expressed from a single plasmid vector for dual expression of reporters. (B) Activity of the *PEG-3* promoter was compared among human lung carcinoma cell line (H460) and human primary normal cells listed above. Each cell were transfected with pPEG-HSV1-tk plasmid or promoter-less control plasmid. The expression of HSV1-tk was assessed by measuring the uptake level of radioactive substrate of HSV1-tk, [^{125}I]FIAU (1-(2-deoxy-2-fluoro-1-D-arabinofuranosyl)-5-iodouracil).

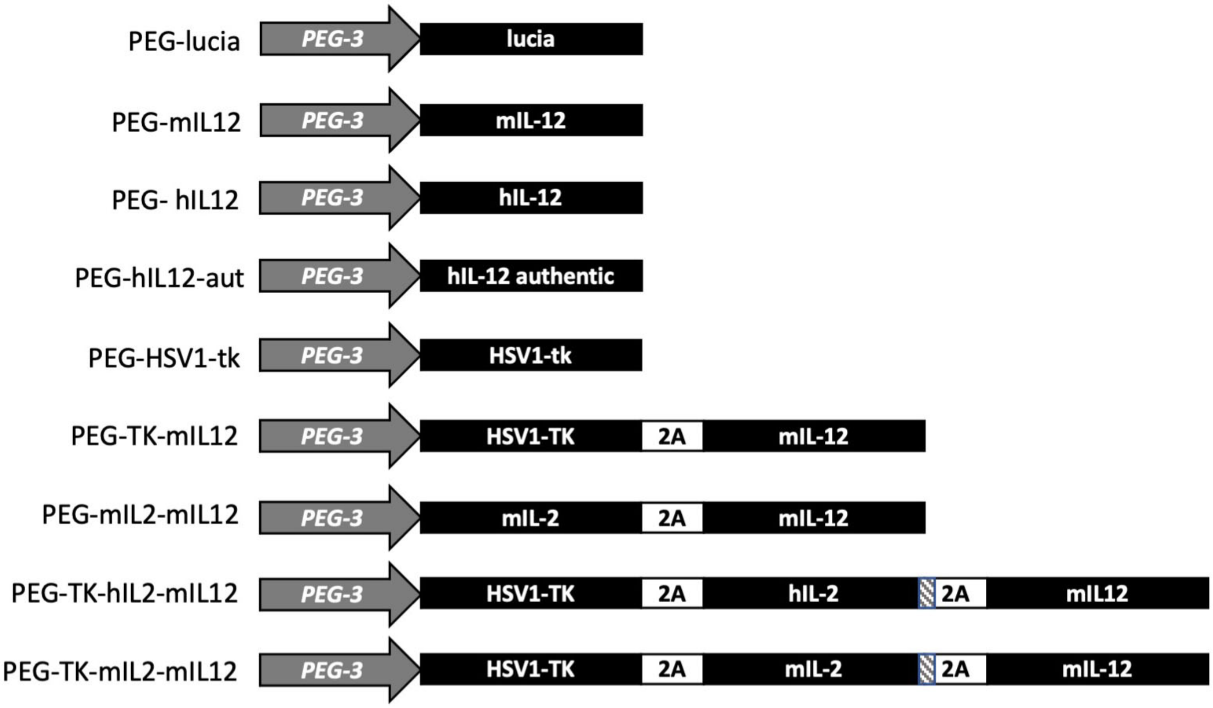

Fig. S2: Diagram of the expression cassettes for the plasmids tested in this study. Each expression cassette is driven by the *PEG-3* promoter, individual protein coding regions were separated by a picornavirus 2A sequence (2A) and additionally a furin cleavage site (hatched box). Each cassette was engineered to eliminate CpG sites except for hIL12-aut, which is authentic hIL-12 cDNA including CpG sequences. Size of plasmids: PEG-TK-mIL12, 6098 bp; and PEG-TK-hIL2-mIL12, 6632 bp; compared with PEG-mIL12, 4898 bp; PEG-HSV1-tk, 4393 bp; PEG-hIL12 and PEG-hIL12-aut, 4885 bp; and PEG-lucia, 3888 bp.

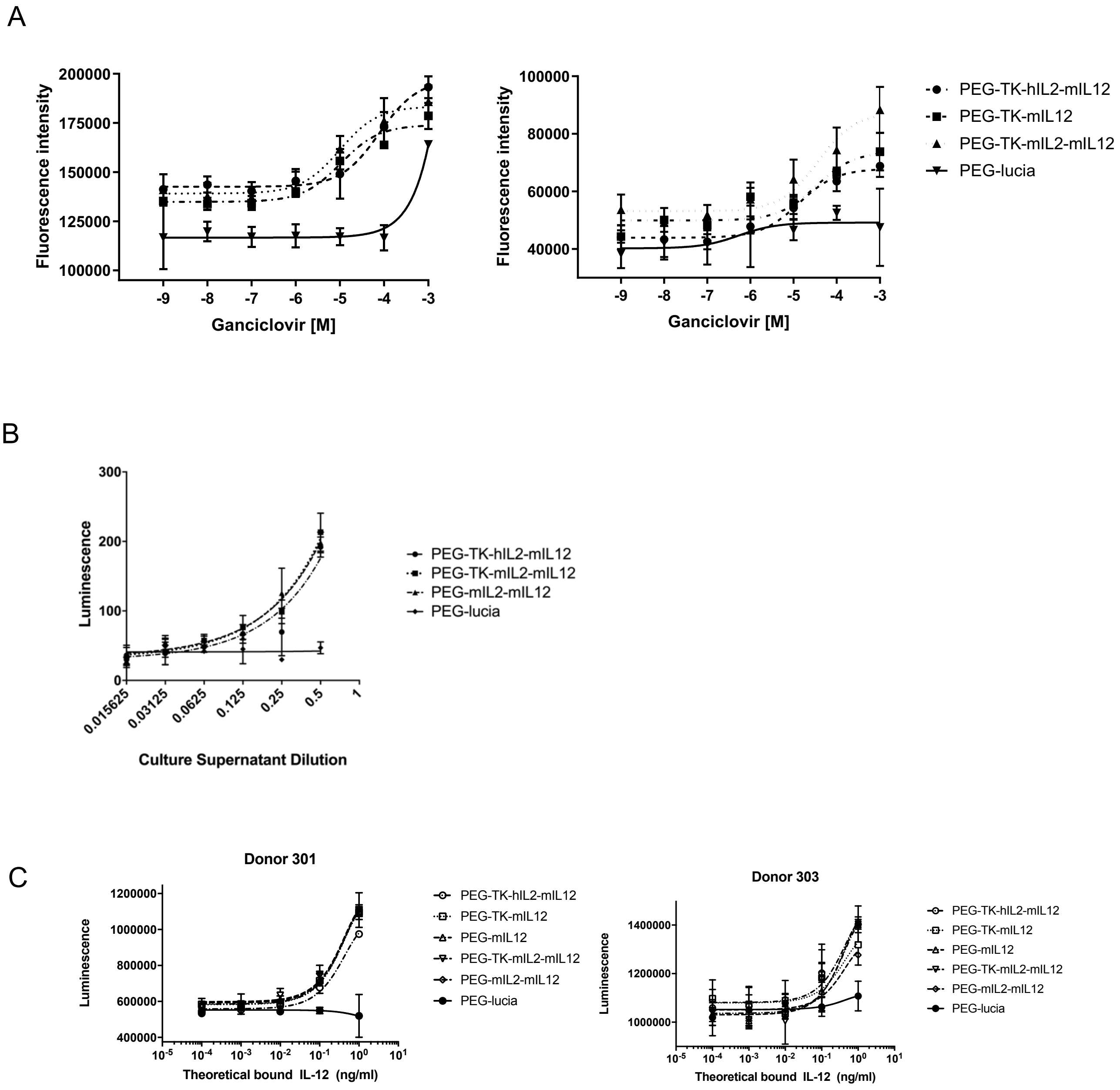

Fig. S3: Functional activity of expressed genes *in vitro*. Activity of the genes expressed from plasmids PEG-TK-hIL2-mIL12, PEG-TK-mIL12, PEG-TK-mIL2-mIL12 and PEG-lucia were evaluated in three different assays. (A) phosphorylation of ganciclovir by plasmid expressed HSV1-TK as assayed by cytotoxicity from two different experiments in triplicate (except PEG-TK-hIL2-mIL12 in the first graph, which was duplicate. (B) CTLL2 proliferation assay to show stimulation of murine CTLL2 T-cells by the expressed genes mIL-12 and h-IL2 (in triplicate for PEG-TK-hIL2-mIL12 and PEG-TK-mIL2-mIL12 but duplicate for PEG-mIL2-mIL12 and PEG lucia. (C) human PBMC proliferation assay following capture of murine IL-12 from cell culture (samples tested in duplicate). Least squares fitting was used to plot the curves.

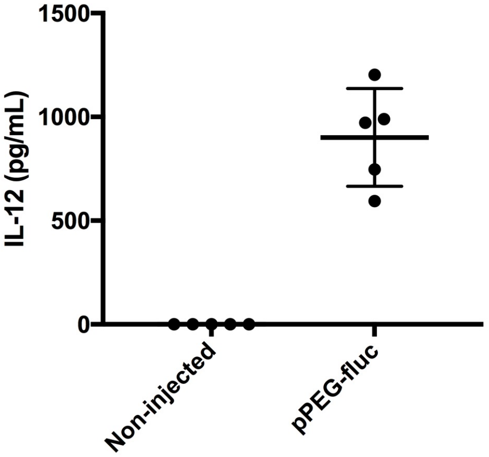

Fig. S4: Induction of significant level of IL-12 via CpG-rich plasmid. The research grade plasmid, pPEG-fluc containing 357 CpG sequences induced significantly high level of IL-12 in serum of CD-1 mice at 2 h post injection of the nanoparticle.

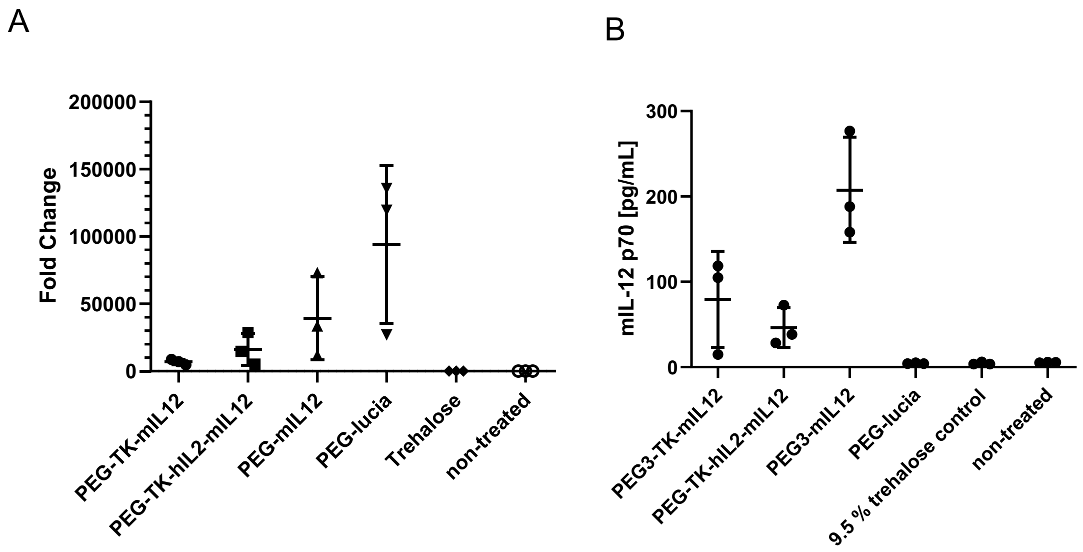

Fig. S5: Quantitative PCR (qPCR) analysis and mIL-12 expression of the plasmid delivery in the lungs of LL/2 bearing mice. (A). qPCR probes were designed to amplify a region of the *PEG-3* promoter to monitor distribution of the plasmid 9 d after tumor cell inoculation and one day following the second treatment with nanoparticles. For this cohort of mice, treatment schedule was 5 and 8 d after tumor cell inoculation rather than 5 and 9 d. (B). expression of mIL-12 in the lungs (following resection of major tumours) as monitored by ELISA. Statistical significance was not calculated for these results due to the limited number of animals (n = 3).

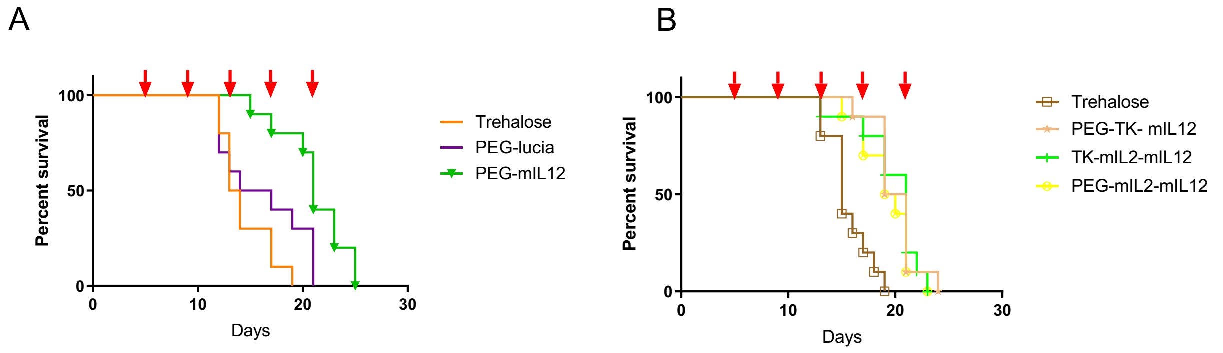

Fig. S6: Activity of l-PEI/DNA nanoparticles in a second study in an LL/2 syngeneic, orthotopic model of primary lung cancer in C57BL/6 mice represented by a Kaplan Meier survival plot. Mice were inoculated orthotopically inoculated with LL/2 Red-FLuc murine lung cancer cell line and treated with nanoparticles at 4-d intervals, beginning on Day 5, as indicated by the arrows above the chart. PEG-mIL12 nanoparticles significantly ($p \leq 0.05$, Log-rank) extended survival in this model compared to the vehicle control (trehalose control) and PEG-lucia control (A). PEG-TK-mIL12, PEG-TK-mIL2-mIL12 and PEG-mIL2-mIL12 significantly ($p \leq 0.05$, Log-rank test) extended survival in this model compared to the vehicle control (9.5% trehalose) (B).

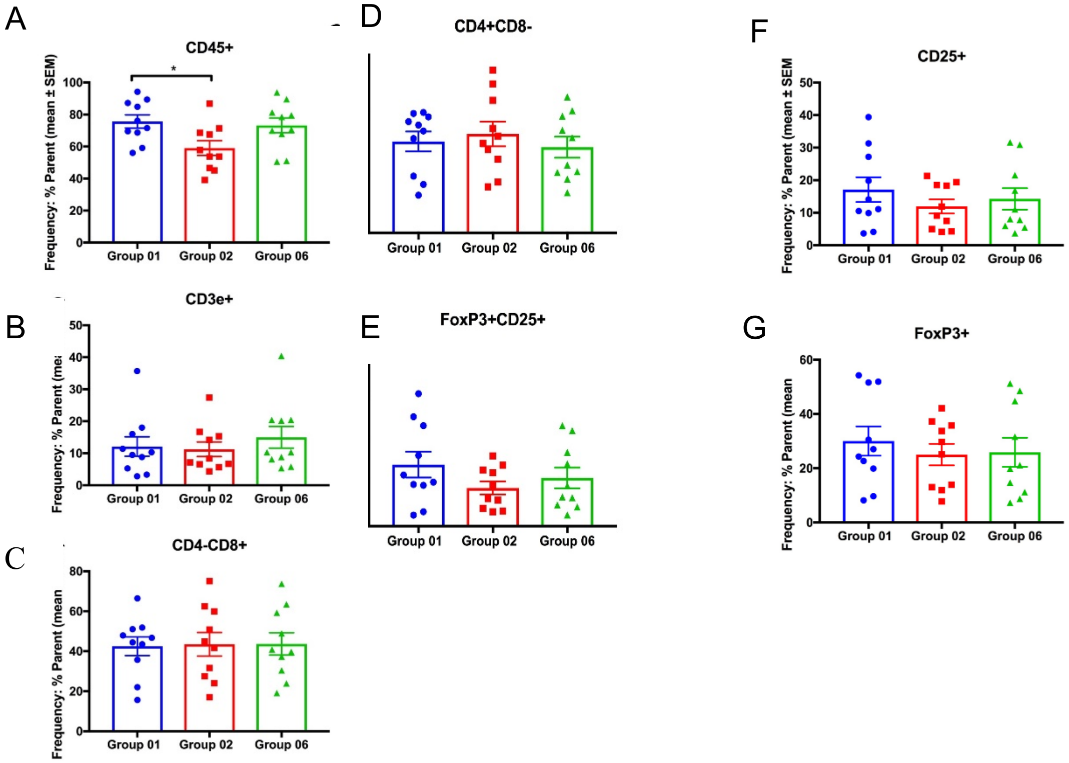

Fig. S7: FACS analyses of the cell populations within the LL/2 tumours in mice at the time of termination. Group 1 animals were the vehicle control (treated with 9.5% trehalose); Group 2 animals were treated with PEG-mIL12; Group 6 animals were in the nanoparticle control group (PEG-lucia). CD45+ lymphocytes were significantly lower in the mice treated with PEG-mIL12 compared to the vehicle control (A). However, there were no significant differences in the populations of CD3ε+ (B), CD4-CD8+ (C), CD4+CD8- (D), FoxP3+CD25+(E), CD25+ (F), FoxP3+ expression levels (G) although the population of T_{reg} cells (E, FoxP3+CD25+) in PEG-mIL12 (Group 2) was less than the controls.

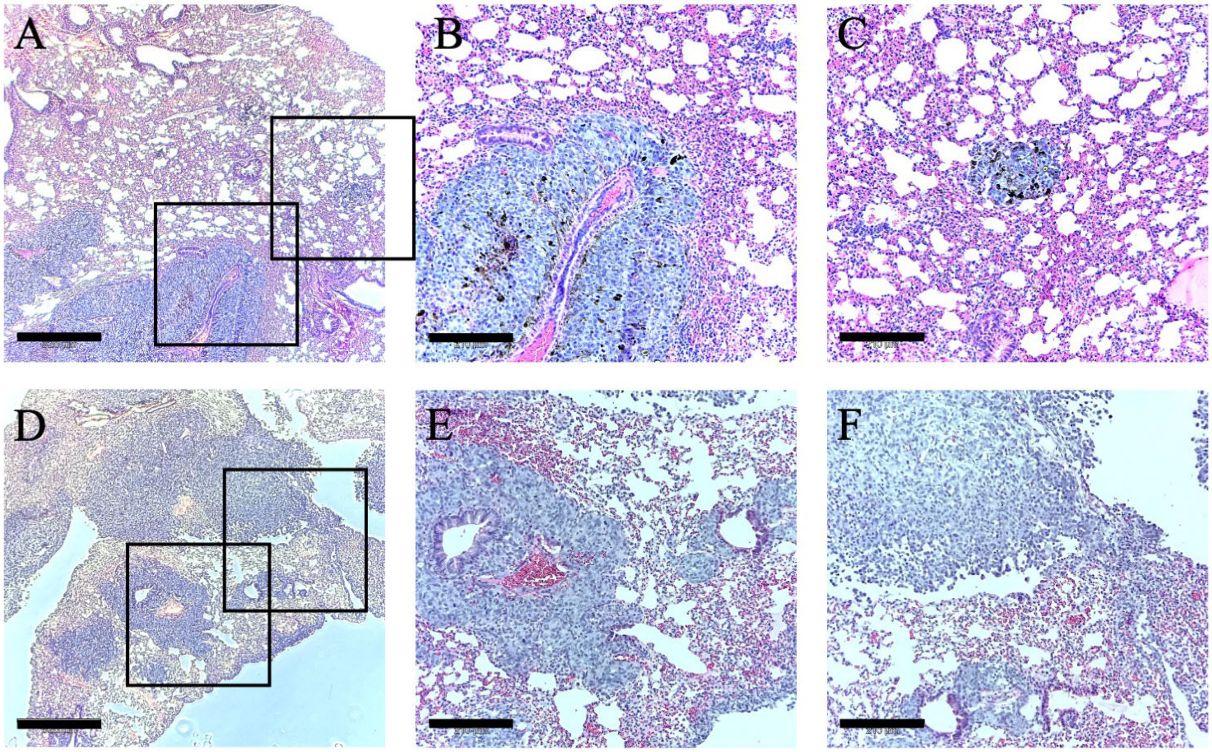

Fig. S8: Histological confirmation of the lung tumor models. (A-C) LL/2-Red-Fluc cells orthotopically injected in a lung of C57BL/6 mice. (D-F) B16F10-Red-FLuc cells intravenously injected in a lung of C57BL/6 mice. H&E stained slides were microscopically captured with 4x objective (A and D, scale bar: 520 μm) and 10x objective (B, C, E, and F) scale bar: 210 μm). Squares in A and D present the enlarged images.

Table S1: Expression of IL-12 and IL-2 in LL/2 Red-Fluc Cells in Culture

PEG Plasmid construct	Mean mIL-2 yield (pg/mL, n = 2)	Mean mIL-2 yield ng/10^6 (48 h, n = 2)
**PEG-mIL2-mIL12**	1107.0 ± 222.0	11.1 ± 2.2
**PEG-TK-mIL2-mIL12**	1556.0 ± 271.0	15.6 ± 2.7
**PEG-lucia**	135.0 ± 81.0	1.3 ± 0.8

The data presented were the expression level of mIL-2 in the cell culture media measured by ELISA.

PEG Plasmid construct	Mean mIL-12 yield (pg/ml, n = 2)	Mean mIL-12 yield ng/10^6/48 h (n = 2)
**PEG -mIL12**	10896.0 ± 43.1	109.0 ± 0.4
**PEG-TK-mIL2**	16465.2 ± 160.0	164.7 ± 1.6
**PEG-mIL2-mIL12**	8460.0 ± 1380.0	84.5 ± 13.8
**PEG-TK-hIL2-mIL12**	15819.1 ± 596.9	158.2 ± 6.0
**PEG-TK-mIL2-mIL12**	10060.0 ± 2910.0	100.6 ± 29.1
**PEG-lucia**	Non detected	Non detected

The data presented were the expression level of mIL-12 in the cell culture media measured by ELISA.

Table S2: Blood markers for liver toxicity, amongst other indicators, compared to control mice

Group	Animal ID	ALP (U/L)	AST (U/L)	ALT (U/L)	Creatine kinase (U/L)	Albumin (g/dL)	Total Bilirubin (mg/dL)	Total Protein (g/dL)	Globulin (g/dL)
1	1450340	61	219	102	301	2.5	0.2	4.4	1.9
	1454350	68	121	52	89	2.4	0.1	4.1	1.7
	1481194	76	161	66	78	2.3	0.2	4.0	1.7
2	1442078	68	141	71	71	2.3	0.2	3.9	1.6
	1458383	63	194	60	209	2.3	0.2	4.0	1.7
	1481946	68	140	61	87	2.5	0.2	4.3	1.8
3	1456800	50	135	52	234	2.3	0.2	4.1	1.8
	1464378	72	237	104	278	2.3	0.2	4.0	1.7
	1515026	59	234	111	137	2.1	0.2	3.8	1.7
4	1453174	58	115	50	70	2.3	0.1	4.1	1.8
	1465142	50	155	43	209	2.4	0.2	4.2	1.8
	1542998	45	147	63	167	2.5	0.2	4.2	1.7
5	1457923	98	109	36	182	3.0	0.2	4.7	1.7
	1493364	65	80	38	91	2.9	0.2	4.6	1.7
	1495315	102	90	32	177	2.9	0.2	4.6	1.7
6	1433640	81	79	32	106	3.1	0.2	5.0	1.9
	1449564	84	126	37	1371	2.9	0.2	4.7	1.8
	1458371	83	75	29	119	3.0	0.1	4.7	1.7

ALP: alkaline phosphatase; AST: aspartate amino transferase; ALT: alanine amino transferase

Group	Animal ID	Bilirubin - Conjugated (mg/dL)	BUN (mg/dL)	Creatinine (mg/dL)	Cholesterol (mg/dL)	Glucose (mg/dL)	Calcium (mg/dL)	Phosphorus (mg/dL)	Bicarbonate TCO_2 (mmol/L)
1	1450340	0.0	20	0.0	93	184	9.3	6.2	26
	1454350	0.0	18	0.1	92	190	9.2	6.5	25
	1481194	0.0	18	0.1	90	237	9.2	7.0	18
2	1442078	0.0	18	0.1	82	240	9.2	8.7	27
	1458383	0.0	21	0.1	91	225	9.2	7.8	28
	1481946	0.0	19	0.1	90	220	9.1	7.0	20
3	1456800	0.0	11	0.1	87	234	8.9	6.8	23
	1464378	0.0	15	0.1	85	215	9.0	6.7	27
	1515026	0.0	14	0.1	90	233	9.1	7.8	23
4	1453174	0.0	13	0.1	100	232	9.1	5.9	28
	1465142	0.0	14	0.0	96	216	8.8	6.8	24
	1542998	0.1	13	0.1	90	244	9.4	6.4	27
5	1457923	0.0	19	0.1	87	283	9.3	6.4	27
	1493364	0.0	12	0.1	93	272	8.8	6.2	26
	1495315	0.0	14	0.1	87	330	9.4	7.8	19
6	1433640	0.0	22	0.1	70	322	9.4	6.3	28
	1449564	0.0	24	0.0	75	323	9.6	8.4	17
	1458371	0.0	21	0.1	73	387	9.4	8.8	21

BUN: Blood Urea Nitrogen

Group	Animal ID	Chloride (mmol/L)	Potassium (mmol/L)	ALB/GLOB ratio	Sodium (mmol/L)	BUN/Creatinine Ratio	Bilirubin - Unconjugated (mg/dL)	Na/K Ratio	Haemolysis Index	Lipaemia Index
1	1450340	109	5.1	1.3	144	0.0	0.2	28	+	Normal
	1454350	114	5.0	1.4	145	180.0	0.1	29	Normal	Normal
	1481194	115	3.6	1.4	146	180.0	0.2	41	+	Normal
2	1442078	116	3.9	1.4	147	180.0	0.2	38	+	Normal
	1458383	117	4.3	1.4	148	210.0	0.2	34	+	Normal
	1481946	113	4.2	1.4	146	190.0	0.2	35	+	Normal
3	1456800	110	5.1	1.3	144	110.0	0.2	28	+	Normal
	1464378	115	4.6	1.4	145	150.0	0.2	32	+	Normal
	1515026	116	3.7	1.2	146	140.0	0.2	39	+	Normal
4	1453174	112	5.4	1.3	143	130.0	0.1	26	Normal	Normal
	1465142	106	6.0	1.3	141	0.0	0.2	24	++	Normal
	1542998	111	4.3	1.5	144	130.0	0.1	33	Normal	Normal
5	1457923	108	4.3	1.8	143	190.0	0.2	33	+	Normal
	1493364	111	4.4	1.7	142	120.0	0.2	32	+	Normal
	1495315	108	3.2	1.7	143	140.0	0.2	45	+	Normal
6	1433640	111	4.2	1.6	144	220.0	0.2	34	+	Normal
	1449564	no result	no result	1.6	no result	0.0	0.2	no result	+	Normal
	1458371	109	3.3	1.8	144	210.0	0.1	44	+	Normal

ALB/GLOB: Albumin/Globulin ratio; BUN: Blood Urea Nitrogen; Na/K: Sodium/Potassium ratio
+ slightly; ++ moderately; +++ high; No result: Insufficient sample

Group 1, mice treated with PEG-TK-mIL12 nanoparticles; Group 2, mice treated with PEG-TK-hIL2-mIL12 nanopartilces; Group 3, mice treated with PEG-mIL12 nanoparticles; Group 4, mice treated with PEG-lucia nanoparticles; Group 5, Trehalose treated mice; Group 6, untreated mice.

Table S3: Details of necropsy for individual mice treated in the B16F10 experimental

Group and Treatment	Animal ID	Necropsy Findings
Group 1 9.5% Trehalose Control	3475345 *	-
	3481051	Multiple metastases in thoracic cavity ranging from ~3-8mm, large 1-13mm metastasis under liver and behind neck subcutaneously
	3489133	Metastases around ovary and dispersed in abdominal wall
	3505397 *	-
	3507249 *	-
	3507597	Metastases on liver and on snout
	3525474	No abnormal findings
	3558142	Metastases around ovary and dispersed through abdomen
	3565875	No abnormal findings
	3588161	Stomach and bowls appear necrotic (black, no visible metastases). Tumour growth within urethra visible and bladder is very full. Metastases also visible in thoracic cavity (adhered to rib cage and connective tissue)
Group 2 PEG-Lucia	3451312	Metastases in thoracic cavity and diaphragm ~3-6mm, intestinal metastasis ~8-11mm, metastasis under L kidney ~4-6mm
	3470281	No abnormal findings
	3490590	No abnormal findings
	3497422 *	Large ovarian metastasis
	3499019	Several abdominal metastases, large upper abdominal metastasis and intestinal metastasis
	3501434	Small metastases < 3mm & ~3-5mm metastases in thoracic cavity, multiple metastases along intestinal tract ranging ~3mm-13mm
	3510444 *	-
	3516248	Metastases on neck and snout.
	3573116	Small metastases in thoracic cavity and a small metastasis along intestinal tract
	3585713	Small metastases in thoracic cavity, 4-6mm metastasis along intestinal tract
Group 3 mIL12	3451376	Metastases on liver and snout.
	3456547	Metastases in small intestine and neck (lymph node) and rib cage. Inflated small intestine, stomach appears normal
	3475682	Large amorphous mass with the same form as metastases completely intertwined with small intestine and filled entire abdominal cavity
	3481779 *	Animal found dead, necropsy was performed but no samples collected due to autolysing of tissues. one large tumour on upper left flank in subcutaneous space. One large metastasis on lung filled half of a lobe.
	3491364	Metastasis in place of left ovary. Inflated gastrointestinal tract. Metastasis on spleen.
	3498357	Very large submandibular gland right side, metastasis on lower right flank in muscle
	3499982 *	-
	3509328	Metastasis in place of right ovary. Metastases in pelvic fat, mesentery of small intestine, spleen, liver, rib cage and neck
	3562662	black (possibly faeces) in portion of gastrointestinal tract, small mass on right kidney, large right submandibular mass
	3586399 *	-

*Animal was found dead. Necropsy not performed unless within 2 hours of death.

Table S3 (continued)

Group and Treatment	Animal ID	Necropsy Findings
Group 4 TK-mIL12	3470333	No visible metastases elsewhere on body apart from lungs
	3479930 *	No visible metastases elsewhere on body apart from lungs
	3481877	Very small spleen and intestines/stomach appear empty. Metastasis on left mandible.
	3482693	Large gelatinous pieces of tumour were present in thoracic cavity (not connected to lung). Not included in weight of lung.
	3490425 *	Small ovary metastasis on right side, large metastasis attached to pelvic fat
	3500035	Large metastasis in pelvic fat
	3504626 *	Small metastasis on left ovary
	3506583	Ovarian metastasis. Metastases in small intestine and colon. Vaginal metastasis. Metastases surrounding kidney.
	3507404	Metastasis in place of each ovary. Metastasis on heart and liver
	3523562	No abnormal findings
Group 5 mIL2-mIL1	3470141	Metastases in snout, coming out of mouth. Gastrointestinal tract including stomach inflated with air
	3475123	Metastases found in left hind limb, right fore-limb, left nostril, spleen, and spine in thoracic cavity. Air-filled intestinal tract, light pink liver.
	3475639	Large right ovary, medium mesenteric lymph node tumour, discoloured greyish liver
	3481721	Metastases around oesophagus and in thoracic cavity. Metastases in pelvic fat and three lobes in pelvic fat. Metastases in place of each ovary. Metastasis on left kidney. Metastasis on middle left flank within subcutaneous space.
	3493587 *	-
	3496210	Metastases on upper right flank in subcutaneous space, right ovary, 2 in pelvic fat, all very large. Small metastasis on right kidney, black necrotic intestines, discoloured greyish liver
	3496565	Metastasis on ventral portion of neck. Metastasis in place of left ovary.
	3497851 *	One large ovarian metastasis, digestive tract was mildly inflated
	3500081	Metastases present on small intestine
	3511772	Metastases filling thoracic cavity. Large tumour found in pelvis, metastasis on right kidney
Group 6 TK-mIL2-mIL12	3473669	Several abdominal metastases saved
	3475099	Large red tumour on left mandible. Metastases in pelvic fat
	3477074	No visible metastases elsewhere on body apart from lungs
	3477866	~20 metastases within mesentary of small intestine. Metastases in pelvic fat mass. Metastasis in axial portion of right fore-limb. Very large metastasis in place of right ovary. Multiple metastases within each lobe of the liver. Very large red/black mass surrounding left kidney.
	3484561	Large abdominal tumour, possibly connected to liver, mesenteric ln tumour, large tumour possibly connected to pelvic fat or urethra
	3485162	Metastases on each ovary. Large metastasis on rib cage.
	3492330	Metastases in place of kidney. Metastasis on liver.
	3493833	Metastases in pelvic fat and filling thoracic cavity. Vaginal tumour present.
	3497256	Very large subcutaneous metastasis on the lower ventral abdomen just above the bladder. Large metastases in place of left ovary. Large metastases in thoracic cavity just above diaphragm connected to the dorsal rib cage-was not attached to lung, thus not included in lung weight. One liver metastases ~3mm.
	3510151	Tumour in place of left ovary with many smaller lobes connected. Large metastases of hilum of right kidney.

*Animal was found dead. Necropsy not performed unless within 2 hours of death.
